# Evaluation of human-papillomavirus screening for cervical cancer in China’s rural population

**DOI:** 10.7717/peerj.8152

**Published:** 2019-12-20

**Authors:** Ling Li, Ziwen Zheng, Longyu Li

**Affiliations:** 1 Nanchang University, Nanchang, China; 2 Jiangxi Cancer Hospital, Nanchang, China; 3 Jiangxi Maternal and Child Hygiene Hospital, Nanchang, China

**Keywords:** Primary Screening, Cervical Cancer

## Abstract

**Background and Objective:**

Human papillomavirus (HPV) testing has become a preferred cervical cancer screening. However, most HPV infections are harmless and additional tests are required to screen HPV positive women. The objective of this study is to determine the optimal triage strategies for HPV positive women in China’s rural population.

**Methods:**

A population-based screening was performed at seven rural counties of Jiangxi province, between October 2014 to January 2016. A total of 18,000 women aged 35–64 years were enrolled in this study. The primary screening was performed using CareHPV, HC-2, Cobas ®4,800 or HybriMax. Positive women were further screened with five triage tests: (1) Liquid-based cytology test (LBC); (2) conventional Pap cytology test (Pap smear); (3) HPV16, 18 detection; (4) viral load; and (5) visual inspection with acetic acid and Lugol’s iodine (VIA/VILI). Women who were tested positive were referred for colposcopy. The five triage tests were compared with respect to sensitivity, specificity, referral rate, cost and diagnostic time.

**Results:**

Complete data were available for 17,782 women. The HPV prevalence was 13.6%. Referral rates for colposcopy were 4.5%, 2.8%, 2.8%, 6.6%, and 3.7% with LBC, Pap smear, HPV16/18, viral load, and VIA/VILI, respectively. The sensitivity of the above triage tests was 65.8%, 51.9%, 86.8%, 73.3%, and 41.7%, respectively. The specificity was 69.8%, 81.0%, 85.8%, 52.2% and 65.3%, respectively. The average time to diagnosis was significantly lower with HPV16/18, viral load and VIA/VILI than LBC and Pap smear. In addition, screening cost that leads to identify one HSIL+ woman was the lowest with viral load.

**Conclusion:**

Our data indicate that HPV16/18 and viral load are the optimal triage strategies for HPV screening in China’s rural population.

## Introduction

Cervical cancer is a leading cause of cancer-related deaths in women (Bray et al., 2018). In developed countries, cervical cancer accounts for 7% of all female cancers, while in developing countries it accounts for 24% of all female cancers (Bray et al., 2018). This disparity is primarily due to the lack of screening and treatment of pre-cancerous lesions (Bray et al., 2018). For example, since the introduction of organized cervical screening in the 1960s, the incidence and mortality from cervical cancer in the United States have declined by 75% (Brensing et al., 2000; Jemal et al., 2011). However, implementation of the same screening program in developing areas is not feasible due to a lack of infrastructure, financial resources, and medical equipment (Basu et al., 2018).

Persistent infection with high-risk HPV is the main cause of cervical cancer (Burd, 2003). The common primary screening tests for cervical cancer include Papanicolaou test (Pap smear), visual inspection of the cervix with acetic acid (VIA), and HPV testing (Burd, 2003). HPV testing has become a preferred screening for cervical cancer in developing countries because it is relatively cheap and easy to perform (Lorincz et al., 2013). In addition, HPV testing can be used to identify women who do not need immediate colposcopy and biopsy because they are infected with low-risk HPV subtypes (Nakamura et al., 2015). The distribution of HPV types varies among geographical regions and populations Human papillomavirus types (Pfister et al., 1983; Huang et al., 1997; Lai et al., 1999). Until now, there are over 100 HPV subtypes, of which, at least 14 are high-risk types that cause cancer (Burd, 2003). Most HPV infections are harmless and additional tests are required to screen the HPV positive women (Liu & Taioli, 2014). Several triage strategies have been developed for HPV screening (Lorincz et al., 2013). However, there is no study to evaluate these triage strategies for HPV screening in China’s rural population. The aim of this study is to determine the optimal triage strategies for cervical cancer screening in China’s rural population.

## Materials and Methods

### Population

**Figure 1:**
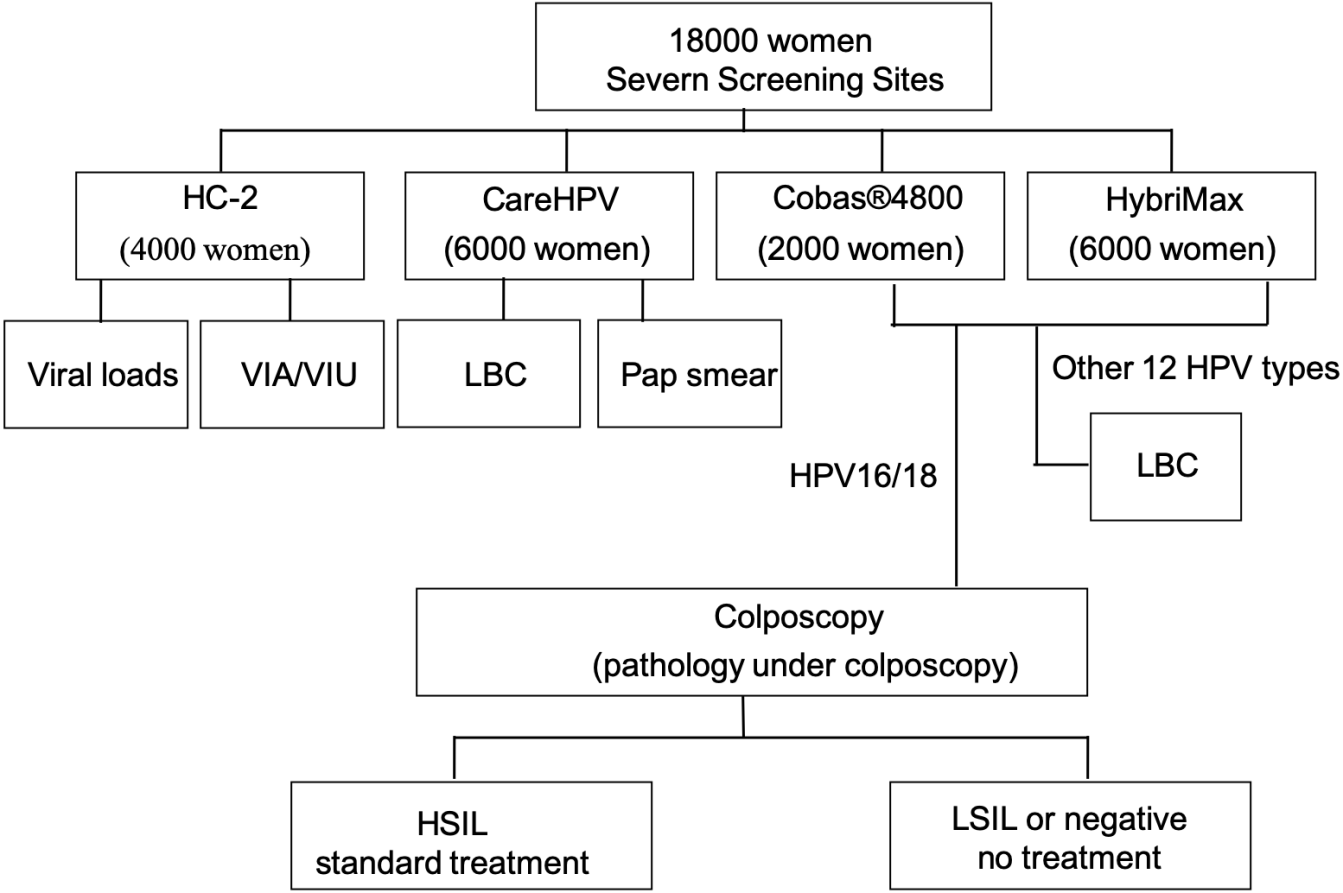
Study flowchart showing the primary HPV screening testing and the triage strategies. HPV, human papillomavirus; VIA/VIU, acetic acid/iodine staining macroscopic observation; LBC, liquid-based cytology; HSIL, high-grade squamous intraepithelial lesion; LSIL, low-grade squamous intraepithelial lesion.

The population-based organized screening was performed between October 2014 to January 2016 in seven rural counties in Jiangxi province, China, including Yushan, Wuning, Xiushui, Leping, Ruijin, Xinyu, and YuShui. Subjects were selected through a stratified, multistage, probability-cluster design. The survey’s components were administered in homes and in mobile examination centers and provided national reference data for biological markers, anthropometric measures, as well as demographic and socioeconomic status data. The eligibility criteria were as follows: women age 35–64 years; sexually active; no history of cervical cancer; not pregnant; have physiologically normal cervix; not screened for cervical cancer within the recent 5 years. Overall, 18,000 eligible women were included in this cohort study. The study protocol (PNC2017076) was approved by the Medical Ethics Committee of Nanchang University. All participants gave their verbal informed consents. The flowchart of the study is shown in Fig. 1.

### The primary HPV screening

The primary HPV screening was performed using multiple tests including careHPV (Qiagen Inc., MD, USA), HC-2 (Qiagen Inc., Frederick, MD, USA), Cobas^®^ 4800 (Roche Molecular System, Inc., Branchburg, NJ, USA), and HybriMax (HybriBio Ltd., Chaozhou, China). The careHPV test contains 8000 base pairs of RNA probes and detects 14 types of high-risk HPV (16, 18, 31, 33, 35, 39, 45, 51, 52, 56, 58, 59, 66, and 68). The results do not indicate the presence of a specific HPV. Samples were considered positive if the RLU/CO was ≥1.00. The HC-2 test is based on the genetic hybridization chemiluminescence-signal amplification technique that detects 13 types of high-risk HPV types (16, 18, 31, 33, 35, 39, 45, 51, 52, 56, 58, 59, and 68). The DNA-RNA hybrids are captured on a microplate, and the emitted light is measured in a luminometer as relative light units (RLU). Samples were considered positive if the RLU/cutoff ratio (CO) was >1.0 (1.0 pg HPV DNA/ml). A RLU/CO value of 1.0–9.99 was defined as low-load. A RLU/CO value of >10.0 was defined as high load. The Cobas^®^ 4800 test is based on real-time PCR and detects 14 types of high-risk type HPV. The test can specifically identify HPV 16/18 subtypes. The HybriMax test is based on HPV nucleic acid amplification and detects15 high-risk HPV types including 16, 18, 31, 33, 35, 39, 45, 51, 52, 56, 58, 59, 68, 73, and 82.

### Triage strategies

Women who were tested positive in the primary screening were further tested by five triage strategies included LBC testing, Pap smear testing, HPV16/18 detection, viral load, and VIA/VILI. Positive women in each triage test were referred for colposcopy. Women with high-grade squamous intraepithelial lesions (HSIL+) received a standard treatment. Women with low-grade squamous intraepithelial lesions (LSIL) or with negative result were not treated.

### Statistical analysis

Statistical analysis was performed using SPSS software version 20.0 (IBM Corporation, Armonk, NY, USA). ROC curves were drawn using Medcalc (Version 16.4.3). The sensitivity and specificity were analyzed with McNemar’s test. The positive and negative predictive values (PPV and NPV) between strategies were analyzed by Chi-square test. A *p*-value of <0.05 was considered statistically significant.

## Results

Complete data were available for 17,782/18,000 (98.8%) women (Fig. 1). The primary screening was performed using CareHPV, HC-2, Cobas^®^ 4800 or HybriMax. The HPV prevalence was 13.56% (2,412/17,782).

A total of 2,981 women were initially screened with careHPV, of which 395 women who were tested positive underwent LBC testing. 134 of them were positive ( ≥ASCUS) and referred for colposcopy. 27 HSIL+ women underwent a cervical biopsy. With this strategy, the colposcopy referral rate was 4.5%. The sensitivity and specificity of this approach was 65.8% and 69.8% respectively. The PPV and NPV were 20.1% and 94.6%. respectively.

A total of 2,899 women were initially screened with careHPV, of which 385 women who were tested positive underwent Pap smear testing. 82 of them were positive ( ≥ASCUS) and referred for colposcopy. 14 HSIL+ women underwent cervical biopsy. With this triage strategy, the colposcopy referral rate was 2.8%. The sensitivity and specificity were 51.9% and 81.0%, respectively. The PPV and NPV were 25.6% and 95.7%, respectively.

A total of 7,910 women were initially screened by Cobas^®^ 4800 and HybriMax, of which 1,152 women were positive. High-risk HPV 16/18 were found in 219 women and referred for colposcopy. 66 HSIL+ women underwent a cervical biopsy. With this strategy, the colposcopy referral rate was 2.8%. The sensitivity and specificity were 86.8% and 85.8%, respectively. The PPV and NPV were 30.1% and 98.9%, respectively.

A total of 2,257 women were initially screened by HC-2. 298 women were tested positive. 104 of them were positive ( ≥10.0 RLU/CO) and referred for colposcopy. 31 HSIL+ women underwent cervical biopsy. With this strategy, the colposcopy referral rate was 2.8%. The sensitivity and specificity were 51.9% and 81.0%, respectively. The PPV and NPV were 25.6% and NPV 95.7%, respectively.

A total of 1,735 women were initially screened with HC-2. 182 women who were tested positive underwent VIA/VILI. 64 of them were positive and referred for colposcopy. 31 HSIL+ women underwent cervical biopsy. With this triage strategy, the colposcopy referral rate was 3.7%. The sensitivity and specificity were respectively 53.3% and 66.5%, respectively. The PPV and NPV were 7.8% and 94.1%, respectively.

**Figure 2:**
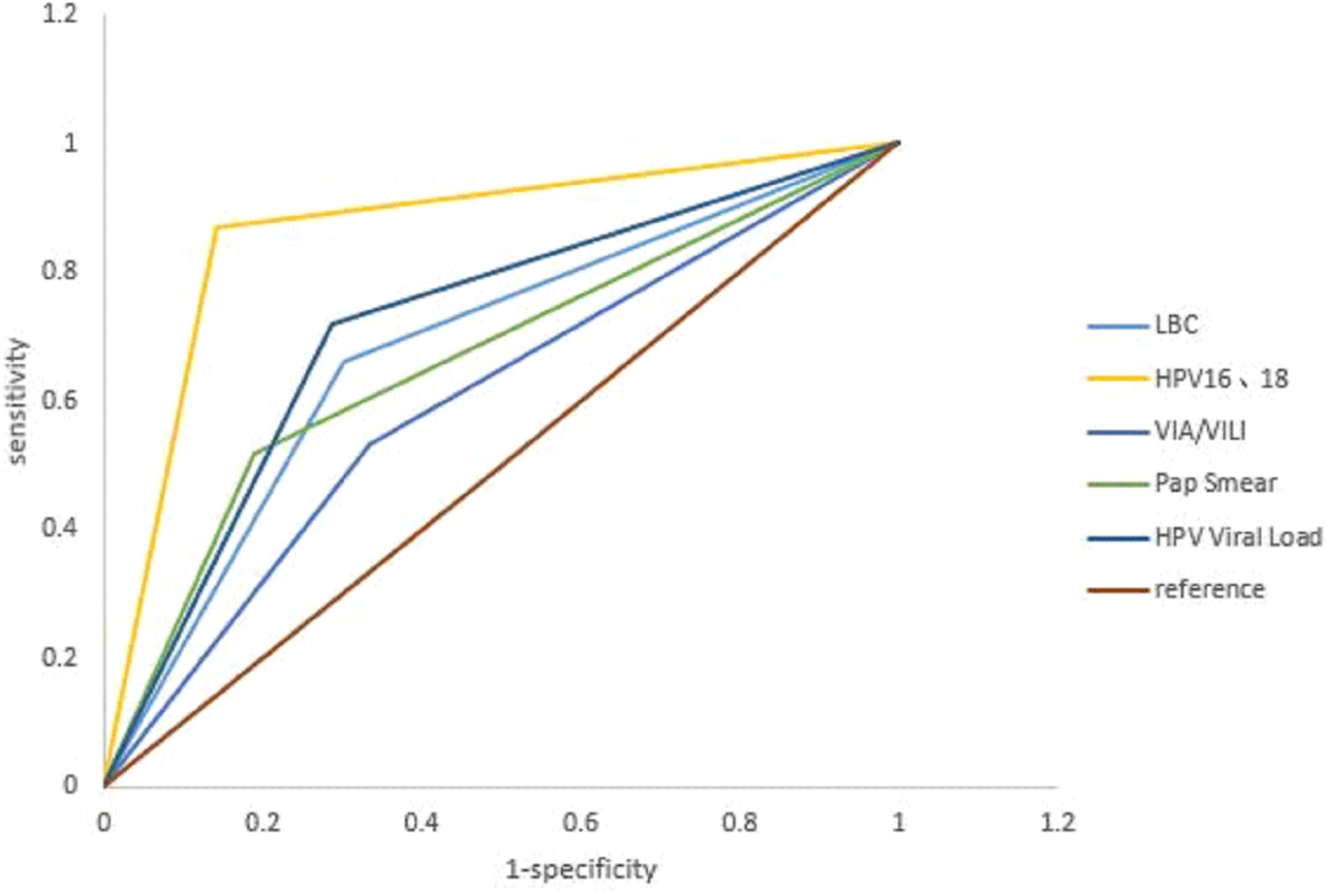
Evaluation of five triage strategies by ROC curve analysis. ROC, Receiver Operating characteristic; HPV, human papillomavirus; VIA/VIU, acetic acid/iodine staining macroscopic observation; LBC, liquid-based.

The sensitivity plotted against 1 −specificity of the five triage strategies were shown in Fig. 2 and the diagnostic accuracy of the five screening strategies was summarized in Table 1. The areas under the curve with LBC, Pap smear, HPV16/18, viral loads and VIA/VILI were 0.678, 0.664, 0.863, 0.717 and 0.599, respectively, suggesting that HPV16/18 is the best option in terms of sensitivity and specificity.

**Table 1:** Diagnostic accuracyof the five screening strategies.

	Sensitivity%	Specificity%	PPV%	NPV%	ROC area
	(95% CL)	(95% CI)	(95% CI)	(95% CI)	(95% CI)
LBC	65.9	69.8	20.1	94.6	0.678
	(61.12, 70.48)	(65.27, 74.33)	(16.15, 24.05)	(92.37, 96.33)	(0.590, 0.767)
Pap	51.9	81.0	25.6	95.7	0.664
Smear	(46.91, 56.89)	(77.08, 84.92)	(21.24, 29.96)	(93.67, 97.37)	(0.584, 0.780)
HPV 16/18	86.8	85.8	30.1	98.9	0.863
	(84.55, 88.75)	(83.78, 87.82)	(27.45, 32.75)	(98.30, 99.50)	(0.818,0. 909)
Viral load	72.1	71.4	29.8	93.8	0.717
	(68.28, 78.32)	(66.27, 76.53)	(24.61, 34.99)	(91.06, 96.54)	(0.633, 0.801)
VIA/	53.3	66.5	7.8	94.1	0.599
VILI	(46.05, 60.55)	(59.64, 73.36)	(3.90, 11.70)	(90.68, 97.52)	(0.446, 0.752)

**Notes.**

PPV positive predictive value NPV negative predictive value ROC Receiver Operating characteristic HPV human papilloma virus VIA/VILI acetic acid/iodine staining macroscopic observation

The comparison of cost and diagnostic time of the five triage strategies was summarized in **Table 2**. LBC and Pap smear required two follow-ups. Their average time to diagnosis was ∼15 days. In contrast, HPV16/18, viral load and VIA/VILI only required one follow-up. Their average time to diagnosis was ∼9 days. The referral rates to colposcopy of were 4.5% and 2.8%, respectively for LBC and Pap smear while they were 2.8%, 2.8% and 3.7, respectively for HPV-16/18, viral load, and VIA/VILI. Therefore, with respect to screening cost that leads to identify one HSIL+ case and diagnostic time, viral load was the best choice.

**Table 2:** Cost and diagnosis time of the five screening strategies.

Triage strategies	Number of follow-ups	Cost per cervical screening (RMB)	Cost per HSIL+ (RMB)	Diagnosis time (days)
LBCI	2	84.9	9382.2	15.7
Pap smear	2	76.8	15918.8	15.5
HPV 16,18	1	65.2	7219.8	9.2
Viral Load	1	67.7	6943.2	9.2
VIA/VILI	1	59.3	200582.0	9.2

**Notes.**

HSIL high-grade squamous intraepithelial lesion and above lesions LBC liquid-based cytology HPV human papilloma virus VIA/VILI acetic acid/iodine staining macroscopic observation

## Discussion

Cytology-based screening programs remain the mainstay of cervical cancer prevention worldwide. They have been demonstrated to reduce cervical cancer incidence and mortality, particularly in developed countries (Louvanto et al., 2014 & Beal et al., 2014). However, the success of these programs relies on infrastructure, financial resources and medical equipment that are often not available in developing countries. HPV is the most important risk factor for cervical cancer. But most HPV infections do not cause cancer. The majority of HPV-associated cancer cases are related to oncogenic mucosal high-risk HPV subtypes (16, 18, 31, 33, 35, 39, 45, 51, 52, 56, 58, 59, and 68) (Burd, 2003; Clifford et al., 2003). As a matter of fact, HPV 16, 18, 31, 33 and 45 cause more than 99% of cervical cancers (Burd, 2003; Clifford et al., 2003). A number of large-scale randomized controlled trials have shown that HPV testing as a primary screening test can detect approximately 50% more high-grade lesions than the Pap test (Cuzick et al., 2006). Therefore, identification of the high-risk HPV subtypes is a potential way for cervical cancer screening.

There have been several studies assessing the utility of HPV testing in cervical cancer screening (Zuna, Moore & Dunn, 2001; Howard et al., 2008; Bhatla & Moda, 2009; Lui, 2013). One study compared the HPV genotypes and viral load among different sites of the genital tract. It found that high-risk HPV genotypes were fairly equivalent across different genital sites but viral loads were largely variable arguing for the necessity of evaluating those screening strategies in a specific population (Zhang et al., 2014b; Zhang et al., 2014a). Another study that compared the diagnostic accuracy of cytology and VIA/VILI to triage HPV positive women found that both methods had the same lesion detection ability (Muwonge et al., 2014). In our study, we evaluated five strategies for HPV screening in rural Chinese population and identified HPV16/18 and viral load to be the optimal strategies because of their low cost, short diagnostic time and good sensitivity and specificity (Tables 1–2; Fig. 2).

Triage strategies for HPV positive women in low- and middle-income countries depend on local health resources, financing, and availability of services. Cytology triage needs a professional and reliable quality control system. According to a study in rural areas in 2014, the effect of viral loads is similar to HPV16/18 (Zhang et al., 2014b; Zhang et al., 2014a). These HPV testing are feasible in developing areas with low resources. Our study evaluated the effectiveness of five feasible triage strategies for HPV testing in less developed areas. The results showed that the sensitivity of LBC and traditional pap triage were not high (65.8% and 51.9%) enough. Both strategies take longer and cost more than the other three triage strategies. The VIA triage costs less and does not need complex equipment and is easy to implement in low-income populations, but the diagnosis is subjective and its sensitivity was relatively low (41.7%). This is consistent with another study showing that VIA triage helps to increase specificity but has low sensitivity (Wang et al., 2017). In addition, the PPV of VIA triage was only 7.8% making it unsuitable for massive screening (Wang et al., 2017). In contrast, the sensitivity of HPV16/18 and viral loads triage was significantly higher (86.8% and 73.3%) and only required one follow-up. The average diagnostic cost was significantly lower than the other three triages ( *P* < 0.05).

There are several limitations to this study. First, although we excluded women with HPV vaccination in this study, we could not completely control the accuracy of the self-reported information. However, the participants in this study, aged 35-64 years, are not recommended for HPV vaccination. Second, the accuracy and performance of the triage testing at different centers may vary among different rural areas. Third, we used colposcopy instead of cytology and histology for diagnosis of HSL, which may underestimate the actual number of HSIL.

## Conclusion

To conclude, we evaluated five different triage strategies for HPV positive women. Our data indicate that HPV 16/18 and viral load are the optimal triage strategies for HPV screening in China’s rural population.

##  Supplemental Information

10.7717/peerj.8152/supp-1 Data S1 Raw data

## Additional Information and Declarations

### Data Availability

The following information was supplied regarding data availability: The data is available as a Supplemental File.
